# On Preventive Medicine, as Illustrated in the Proper Use of Food

**Published:** 1865-11

**Authors:** 


### ON PREVENTIVE MEDICINE, AS ILLUSTRATED IN THE PROPER USE OF FOOD.

Mr. Wilson thinks that we are all, especially our children and youths, much under-fed. He recommends three ample meals of mingled animal and vegetable food; and will have "no putting off of the stomach with bread and butter and slop as the effigies of two of the three meals of the day." Mr. Wilson says:

"But a period comes when milk is no longer the diet of children, and when custom, originating, as we have seen, in Nature's promptings, has determined the necessity of three meals in the day. The infant demands more than three meals, and makes no distinction between the day and the night. The day of the infant is a day of twenty-four hours; the day of childhood, as of the remainder of life, has a duration of twelve to sixteen hours. The three meals at present under consideration are the morning meal, the mid day meal, the evening meal. These meals represent the wants of the body arising during the intervening intervals. The morning meal is intended to supply the moderate waste of the night, the midday meal the active waste of the morning, the evening meal the active waste of the afternoon. The amount of the three periods of waste is pretty equal; the amount of the supply should be equivalent to that of the waste.

"I am desirous of impressing upon my hearers my opinion and firm conviction that food is not only a necessity, but in civilized life a three-fold necessity, and that the three meals should each represent the third of the nourishment of the day, and be so apportioned as to comprehend an equal amount of variety and an equal amount of nourishment. In the primitive life of the laboring class this law is fully appreciated, and is acted upon to the full extent of their means. With the exception of a somewhat more bulky mid day meal, the morning meal and the evening meal do not far diverge from the standard of the mid-day repast.

"But the educated classes are apt to fancy that they possess a knowledge superior to that of Nature, and the result is a perversion of the law of nourishment that leads to the development of debility and disease. A careful, well meaning mother, from purest ignorance—another expression for superior knowledge, the "little" knowledge that is so proverbially dangerous—will tell you that she conforms to the law of Nature in providing for her children three meals in the day. She will describe those meals as breakfast, dinner, and tea, and you will find the composition of those meals to be as follows: A vegetable breakfast, namely, bread and butter, with tea and a little milk; a dinner half animal and half vegetable; and a "tea," vegetable like the breakfast. Here, then, we find education bringing about a total change in the diet of man. Born an animal feeder, he is quickly transformed into a vegetable feeder: that is, more than two-thirds of his diet is vegetable and the remaining third only animal, the exact opposite of that which I consider should be the standard diet of children, namely, one-third vegetable and two-thirds animal.

"My deduction from these premis s is, that children are almost universally under-fed, and that the majority of the diseases of children arise from the debility of constitution induced by this habit of under-feeding. If I am right in this view, preventive medicine may do much in the prevention of disease by correcting an error so widely spread.

"The diet of children of all ages should be, a substantial breakfast, with animal food in some shape; a substantial dinner of meat, vegetables, and cereal pudding; and a substantial supper, also consisting, in part, of animal food. The drink may be milk, tea, cocoa, and, possibly, beer. I would call this the diet of health; a diet capable of making a strong body and also a strong mind; and a diet capable of preventing disease. Compare it for an instant with the milk-and-water and bread-and-butter diet of some establishments: the meagre dinner of meat, and the miserable grouting of rice and amylaceous pulp. Rice and amylaceous pulp should have no place in the diet of health, but should be reserved for the sick room.

"Born in prejudice and matured in prejudice, it is the struggle of a lifetime to throw off the trammels of prejudice. We are apt to attach a peculiar signification to the terms which we are in the habit of employing. Ask a person what he usually takes for breakfast, and he will pretty certainly begin his enumeration with the word "tea," the mere drink of the meal; it is, in truth, with him a mere break-fast, instead of being, as i ought to be, a substantial morning meal. The dinner of laber is the luncheon of fashion; then follows the mildly alkaline and stimulating drink that is termed "the tea;" and last of all comes the supper, the late dinner of fashionable life. We have, therefore, before us a succession of three meals and an intermediate drink, but the drink precedes the last meal; and, therefore, the orderly matron, who is more attentive to her 1, 2, 3 than she is to the intention of the daily fare, prescribes for her children breakfast, dinner, and tea—two slops and a meal. But let her, in good English phrase, call the children's meals breakfast, dinner, and supper, and then we immediately obtain two dinners and one slop, the breakfast—an obvious improvement. I have secured to many a child a reasonable evening meal by suggesting to the mother the mere use of the word "supper" as the name of the third meal. No human being could call bread and butter and tea by the hearty name of supper.

"Assuming that the amount and richness of the supply of food should be determined by the offices which it has to perform, there is no period of life when more food is required than in childhood and youth. The hard-worked laborer in a long summer's day scarcely exhausts a greater quantity of nutritious matter than a growing boy of ten or twelve years of age; in the laborer the consumption is waste; in the growing boy it is bestowed in the construction of the body, in developing and building up the future man. And it is no uncommon thing to find that although the general construction of the body has been fairly performed, there is some one organ of the economy that has fared less well than the rest, and that part not uncommonly the skin; hence the origin of acne, of the ringworms, *et hoc genus omne*.

"If it be admitted that food is the source of the elements of which the body is composed, what kind of body can be expected in the case of a deficient supply of food, whether that deficiency proceed from actual want or from some perverse theory of refinement founded on a false conception of the nature and objects of food, and of its direct convertibility into the flesh and blood of man? Parents are too apt to take their own stomachs as the standard of diet of their children; a cup of tea and a slice of toast suffices for them, so it must suffice for the little ones. I knew a lady who brought up her children on mutton alone, because she herself could digest nothing but mutton. Her children were a feeble, puny, sheepish race, always in the doctor's hands. A mother, in anticipation of the full meal at seven o'clock, can afford a light lunch; but she unfortunately concludes that, because a light mid-day meal is good for her, a spare dinner is equally proper for her children. She has heard somewhere that suppers are heavy and interfere with sleep; so that the children must be content with their tea, and go supperless to bed. Parents have rights over their children, but not the right of feeding them in such a manner as to make them the subject of disease. Such parents become the authors of a puny and degenerate race, and are unintentionally traitors to their country,

"If the two periods of life already adverted to be important in in their influence on the future man—namely, the period of infancy, ranging from birth to the age of two years, and the period of childhood, ranging from two years to seven years —the next two periods, namely, those of boyhood and youth, are equally so. While the food of the infant and the food of the child are abundant and regular, the food of the boy and the food of the youth should be the same. Both are occupied in the great business of growing life; on both are dependent the future man, for his strength and for his manhood."—*N. Y. Med. Journal. Half-Yearly Ab. of the Med. Sciences, etc.*

